# Nalmefene attenuates neural alcohol cue-reactivity in the ventral striatum and subjective alcohol craving in patients with alcohol use disorder

**DOI:** 10.1007/s00213-021-05842-7

**Published:** 2021-04-12

**Authors:** Damian Karl, J. Malte Bumb, Patrick Bach, Christina Dinter, Anne Koopmann, Derik Hermann, Karl Mann, Falk Kiefer, Sabine Vollstädt-Klein

**Affiliations:** 1 Department of Addictive Behaviour and Addiction Medicine, Central Institute of Mental Health, University of Heidelberg, Medical Faculty Mannheim, Mannheim, Germany; 2 Feuerlein Center on Translational Addiction Medicine (FCTS), University of Heidelberg, Heidelberg, Germany; 3 Mannheim Center for Translational Neurosciences (MCTN), University of Heidelberg, Medical Faculty Mannheim, Mannheim, Germany

**Keywords:** Harm reduction, Reduced drinking, Pharmacotherapy, Controlled drinking, Opioid receptors, Alcohol cue-reactivity, Striatum

## Abstract

**Rationale:**

Alcohol use disorder is a common and devastating mental illness for which satisfactory treatments are still lacking. Nalmefene, as an opioid receptor modulator, could pharmacologically support the reduction of drinking by reducing the (anticipated) rewarding effects of alcohol and expanding the range of treatment options. It has been hypothesized that nalmefene acts via an indirect modulation of the mesolimbic reward system. So far, only a few imaging findings on the neuronal response to nalmefene are available.

**Objectives:**

We tested the effect of a single dose of 18 mg nalmefene on neuronal cue-reactivity in the ventral and dorsal striatum and subjective craving.

**Methods:**

Eighteen non-treatment-seeking participants with alcohol use disorder (67% male, *M* = 50.3 ± 13.9 years) with a current high-risk drinking level (*M* = 76.9 ± 52 g of pure alcohol per day) were investigated using a cue-reactivity task during functional magnetic resonance imaging (fMRI) in a double-blind, placebo-controlled, cross-over study/design. In addition, self-reported craving was assessed before and after exposure to alcohol cues.

**Results:**

An a priori defined region of interest (ROI) analysis of fMRI data from 15 participants revealed that nalmefene reduced alcohol cue-reactivity in the ventral, but not the dorsal striatum. Additionally, the subjective craving was significantly reduced after the cue-reactivity task under nalmefene compared to placebo.

**Conclusion:**

In the present study, reduced craving and cue-reactivity to alcohol stimuli in the ventral striatum by nalmefene indicates a potential anti-craving effect of this drug via attenuation of neural alcohol cue-reactivity.

**Supplementary Information:**

The online version contains supplementary material available at 10.1007/s00213-021-05842-7.

## Introduction

Alcohol use disorder (AUD) is one of the most prevalent substance use disorders worldwide (Peacock et al. 2018). However, only 22% of AUD patients in Europe receive an addiction-specific treatment (Rehm et al. 2015). One possible reason for this low treatment rate could be a lack of willingness for abstinence. Even if abstinence should be the primary goal of addiction treatment, reducing alcohol consumption as a harm reduction may be an alternative treatment option. Against this background, reducing this unwanted treatment gap might be achieved by offering patients to choose between abstinence and reduced drinking as their individual treatment goal (Ambrogne 2002; Batra et al. 2016; Sobell and Sobell 1995).

Treating alcohol use disorder pharmacologically, disulfiram (approved by U.S. Food and Drug Administration [FDA] but not approved by European Medicines Agency [EMA] anymore), acamprosate (approved by FDA and EMA), as well as naltrexone (approved by FDA and EMA), and nalmefene (approved by EMA) present the currently available and approved options (Soyka and Müller 2017). Naltrexone, which was indicated for relapse prevention, to remain abstinent and to reduce craving in alcohol use disorder (Anton 2008) is a (μ-, δ-, and κ-) opioid receptor antagonist (Hendershot et al. 2016). By this means, opioid antagonists play a key role in mediating the rewarding effects of alcohol (Gianoulakis 2001), by suppressing the alcohol-induced release of dopamine in the mesolimbic reward system (Spanagel and Weiss 1999; Spanagel and Zieglgansberger 1997), which again might reduce the subjective alcohol craving (Hendershot et al. 2016; O'Malley et al. 1992; Volpicelli et al. 1992). The approval by the FDA was based on these data showing that naltrexone decrease relapse rates to alcohol use, reduced drinking days, and alcohol craving (O'Malley et al. 1992; Volpicelli et al. 1992) probably by reducing the positive reinforcing effects of alcohol and/or the anticipation of such effects (Heilig et al. 2010). This is also reflected in a decreased fMRI cue-reactivity by naltrexone in the ventral striatum of non-treatment-seeking alcoholics (Myrick et al. 2008). The ventral striatum has been associated to motivational reward processes, which is manifested in a greater activation by alcohol respectively reward-associated stimuli, while the dorsal striatum has been linked to stereotyped and automated behavior (Braus et al. 2001; Everitt and Robbins 2005; Everitt and Robbins 2016; Schacht et al. 2013). Also, imaging studies indicate that there is a shift from ventral to dorsal cue processing (Vollstädt-Klein et al. 2010) and overreliance on habitual learning with increased activation in dorsal striatum in the presence of alcohol dependence (Sjoerds et al. 2013) in the course of addiction development. Against this background, cue-induced brain activation measured with fMRI in the striatum and the ACC was associated with an increased amount of drinking at follow-up (Courtney et al. 2016; Grüsser et al. 2004). In addition to these neurobiological findings, a meta-analysis of Jonas et al. (2014) showed that the numbers needed to treat for benefit (NNTs) for naltrexone (50 mg/day) were 20 to prevent return to any drinking and 12 to prevent return to heavy drinking. Considering this, recently, nalmefene has been approved by the EMA specifically for the reduction of alcohol consumption in adult patients suffering from AUD and a high drinking risk level (> 60 g pure alcohol on a single drinking day for men and > 40 g for women), without physical withdrawal symptoms and not requiring immediate detoxification (Online document. Online document. European Medicines Agency [EMA] 2013). In 2005, about 11 million people in Europe aged between 18 and 64 years suffered from alcohol dependence, and in 2009, 15% of men and 8% of woman consumed alcohol at a high or very high risk level in Europe (Rehm et al. 2012) according to which nalmefene would address a sizeable group.

Nalmefene acts as an opioid system modulator with antagonistic activity at the μ- and δ-receptors, as well as naltrexone, and partial agonistic activity at the κ-receptor (Bart et al. 2005), distinguishing the drug from other drugs that act within the opioid system, such as naltrexone which is a full kappa receptor antagonist (Swift 2013; Vollstädt-Klein et al. 2019).

Besides this different pharmacological principle, the treatment with nalmefene also differs from previous treatment strategies. While therapy with naltrexone takes place continuously, nalmefene should be taken as needed before high-risk drinking situations (Online document. Online document. European Medicines Agency [EMA] 2013). On this occasion, nalmefene should reduce the reinforcing effect of alcohol due to its influence on the mesolimbic reward system, alleviating the reduction of alcohol consumption.

So far, previous investigations showed that nalmefene as on-demand medication is superior to placebo in reducing the number of heavy drinking days (Gual et al. 2013; Mann et al. 2013; Mann et al. 2016; van den Brink et al. 2013). More recent investigations have also shown that nalmefene given as needed reduces the number of heavy drinking days after 12 weeks compared to placebo (Miyata et al. 2019) and treatment as usual (Castera et al. 2018). One further study Quelch et al. (2017) demonstrated that nalmefene reduces in the presence of the alcohol infusion the neuronal response regarding the anticipation of reward in striatal areas if compared to placebo.

Nevertheless, hitherto, the neurobiological mechanism of action of nalmefene is not well understood and needs further investigation. Therefore, we investigated the effect of single-dose nalmefene on neural alcohol-cue-reactivity during fMRI and subjective alcohol craving. We hypothesized that a single dose of nalmefene is superior over placebo in decreasing neural reactivity in the ventral and dorsal striatum, following the presentation of alcohol associated visual stimuli. This would suggest that nalmefene can dampen the hedonistic effects of alcohol or counteract the anticipation of this effect. Due to the shift from ventral to dorsal striatal cue processing (Vollstädt-Klein et al. 2010) and preliminary fMRI findings that show that opiate antagonists like naltrexone and nalmefene are able to reduce brain activity in mesolimbic pathway after cue exposure (Bach et al. 2020; Myrick et al. 2008; Quelch et al. 2017), we concentrate in particular on the ventral and dorsal striatum.

## Methods and materials

The effect of a single-dose nalmefene (18 mg) on cue-reactivity was examined prospectively, using a double-blind, placebo-controlled study design (cross-over design) and functional magnetic resonance imaging (fMRI) (registration at clinicaltrials.gov; NCT02372318). Heavy-drinking (alcohol consumption > 60 g for men and > 40 g for women; at least 5 days/week), non-treatment-seeking participants with alcohol use disorder (≥ five DSM-5 AUD criteria) were selected as the target population. Recruitment was conducted through local notices, several press releases, advertisements, and bulletin in social media. All participants provided written informed consent prior to study participation.

### Study design

**Fig. 1:**
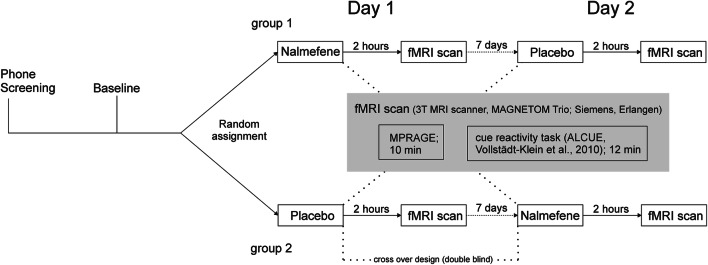
Study procedures

The study consisted of a telephone screening, a baseline screening, and two investigational days including fMRI examination of cue-reactivity (see Fig. 1). Participants had to meet all following inclusion criteria in order to take part in the study: (1) participants had to be aged between 18 and 70 years; (2) they had to meet at least five diagnostic criteria for an alcohol use disorder according to the Diagnostic Statistical Manual of Mental Disorders (DSM-5); (3) the average amount of consumed pure alcohol should be at least ≥ 60 g for men and ≥ 40 g for women per day (at least 5 days/week); (4) they should have a sufficient visual acuity (binocular [corrected] ≥ 0.8). Participants were excluded if they met one or more of the following exclusion criteria: (1) previous inpatient detoxification treatment; (2) current withdrawal symptoms (CIWA-Ar > 4; Sullivan et al. 1989), previous severe withdrawal or any withdrawal complications; (3) other Axis I psychiatric diagnoses than alcohol or tobacco use disorder in the last 12 months screened with Structured Clinical Interview (SKID-I) for DSM-4 (Wittchen et al. 1997) due to the unavailability of a SKID for DSM-5 at the time of examination; (4) neurological disorders respectively history of brain injury; (5) at the time of the examination psychotropic medication within the last 14 days; (6) an intoxication (breath alcohol concentration > 0.3‰); (7) positive drug screening (opioids, cannabinoids, benzodiazepines, barbiturates, cocaine, amphetamines); (8) positive pregnancy test or (9) contraindications to the prescription of nalmefene (e.g., known intolerance, current use of opioid analgesics or opioid-containing antidiarrheal, positive opioid in urine, opiate withdrawal syndrome, or severe liver dysfunction); and (10) exclusion criteria for MRI (e.g., metal implants and claustrophobia).

On screening day, participants were informed about study procedures and possible risks such as medication side effects or headaches after fMRI. Basic sociodemographic information was documented, and history of somatic illnesses and neurological and mental disorders as well as current medication was recorded. Health status was assessed by medical examination. Absence of current illicit drug abuse was verified via urine screening; for women, a pregnancy screen was conducted additionally. An alcohol breath test was performed to confirm abstinence (breath alcohol concentration > 0.3). After medical check-up, participants underwent the Structured Clinical Interview for DSM-4 to verify the absence of Axis-I disorder (SKID-I; Wittchen et al. 1997). This interview was chosen as there was no SKID interview available for DSM-5 at the time of the investigation. Alcohol consumption during the last 3 months was recorded using the Form 90 interview (Scheurich et al. 2005), as well as nicotine status and consumption during the last 3 months. Psychometric assessment comprised the Fagerstrøm Test of Nicotine Dependence (FTND; Heatherton et al. 1991), the Alcohol Dependence Scale (ADS; Skinner and Allen 1982), the Inventory of Drinking Situations (IDS; Annis et al. 1987), the Beck Depression Inventory (BDI; Beck et al. 1961), and the Alcohol Use Disorders Identification Test (AUDIT; Reinert and Allen 2002), recorded via an electronic platform (Social Science Survey, www.soscisurvey.de).

At the investigational day, participants underwent a medical check-up for occurrence of somatic illnesses representing a contraindication for assessment or nalmefene intake. An alcohol breath test was performed to ensure abstinence. Current withdrawal symptoms were captured with the Clinical Institute Withdrawal Assessment for alcohol scale (CIWA-Ar; Sullivan et al. 1989). After assuring the absence of any exclusion criteria valid at MRI investigation, study medication was handed out to the participant, and intake was supervised. Alcohol consumption since baseline measurement or investigational day one respectively was recorded using the Form 90 interview (Scheurich et al. 2005). A second investigational day was scheduled at a 1-week interval and differed only in the double-blind, randomized, oral administration of nalmefene (18 mg) or placebo 2 h before the fMRI measurement. These timeframes were determined on the basis of the pharmacokinetic parameters for nalmefene, namely a time to peak concentration of 0.8 h (median) and opioid receptor occupancy up to 74 h after oral administration of (20 mg) nalmefene (Ingman et al. 2005). Participants were then discharged for a recreational period until a 2-h period after medication intake was fulfilled to ensure substance invasion. Before fMRI measurement, participants’ severity of craving was captured via paper–pencil assessment applying the Alcohol Urge Questionnaire (AUQ, 8 items on a seven-point rating scale; Bohn et al. 1995) and the Alcohol Craving Questionnaire (ACQ, 30 items on a seven-point rating scale; German version, Raabe et al. 2005). Subsequently, participants underwent fMRI measurement comprising (1) our alcohol cue-reactivity task (Vollstädt-Klein et al. 2010) and (2) an emotional faces task, which has been analyzed elsewhere (Vollstädt-Klein et al. 2019). Directly afterwards the fMRI scan, the questionnaires (AUQ, ACQ) were applied one more time. After a quick medical check-up by a physician, participants were dismissed. Test for successful blinding was conducted by asking the participants about their estimation for the time point of the medication administration (56% did not make any estimate, 39% correctly identified it, and 6% made an incorrect estimate).

### Alcohol cue-reactivity task

For the assessment of neural response to alcohol-related stimuli, a cue-reactivity task (ALCUE, Vollstädt-Klein et al. 2010) was used. In this task, 60 alcohol-related and 45 neutral stimuli were presented using a blocked design with five stimuli each block. Each image was presented for 4 s, so each block took 20 s. Alcohol-related pictures were taken from a validated picture series (Vollstädt-Klein et al. 2010), and neutral control cues were taken from the International Affective Picture System. Following each block, the participants were asked for the current intensity of their alcohol craving on a visual analogue scale (VAS) ranging from 0 (no craving) to 100 (extremely extensive craving). Task duration was 12 min.

### Functional MRI acquisition

Functional and anatomical brain images were acquired using a 3T whole-body tomograph (MAGNETOM Trio, Siemens Medical Systems, Erlangen, Germany). Task-related blood oxygen level-dependent (BOLD) response was measured using T2*-weighted echo planar imaging (EPI) sequences (TR = 2.41 s, TE = 25 ms, flip angle = 80°, 42 slices, slice thickness 2 mm, 1 mm gap, voxel dimensions 3 × 3 × 3 mm^3^, FOV 192 × 192 mm^2^, 64 × 64 in-plane resolution). The T2*-weighted EPI sequences were acquired in a transversal orientation 30° clockwise to AC-PC-line covering the whole brain. This short TE and the 30° flip to AC-PC orientation were chosen to minimize susceptibility artifacts. The number of images measured at the ALCUE Task for each participant was 305.

In addition, high-resolution anatomical scans using T1-weighted 3-D magnetization-prepared rapid acquisition gradient-echo (MPRAGE) sequences consisting of 192 sagittal slices (slice thickness 1 mm, 1 × 1 × 1 mm voxel size, FOV 256 × 256 mm^2^, TR = 2300 ms, TE = 3.03 ms, TI = 900 ms, flip angle = 9°) were acquired for each participant.

### fMRI pre-processing

Pre-processing and statistical analyses of brain imaging data were performed using SPM8 (Wellcome Department of Cognitive Neurology, London, UK). The first five scans were excluded from the analyses to avoid artifacts due to magnetic saturation effects. The remaining scans were realigned spatially to correct for head motion over the course of the session and then normalized to an MNI (Montreal Neurological Institute, Quebec, Canada) EPI template. Subsequent smoothing was performed using an isotropic Gaussian kernel for group analysis (8 mm full width at half maximum [FWHM]).

### Statistical analysis

Statistical analyses of the pre-processed fMRI data on the first (individual) level were performed by modeling the different conditions (alcohol-associated versus neutral control stimuli; boxcar functions convoluted with the hemodynamic response function) as explanatory variables within the context of the general linear model (GLM) on a voxel-by-voxel basis with SPM8. Realignment parameters were included as regressors of no interest. The following contrast images were calculated for each participant and each drug condition: (1) favorite drink > neutral; (2) neutral > favorite drink (e.g., [1 0 0 –1] for beer drinkers and [1 1 0 –2] for beer and wine drinkers).

**Fig. 2:**
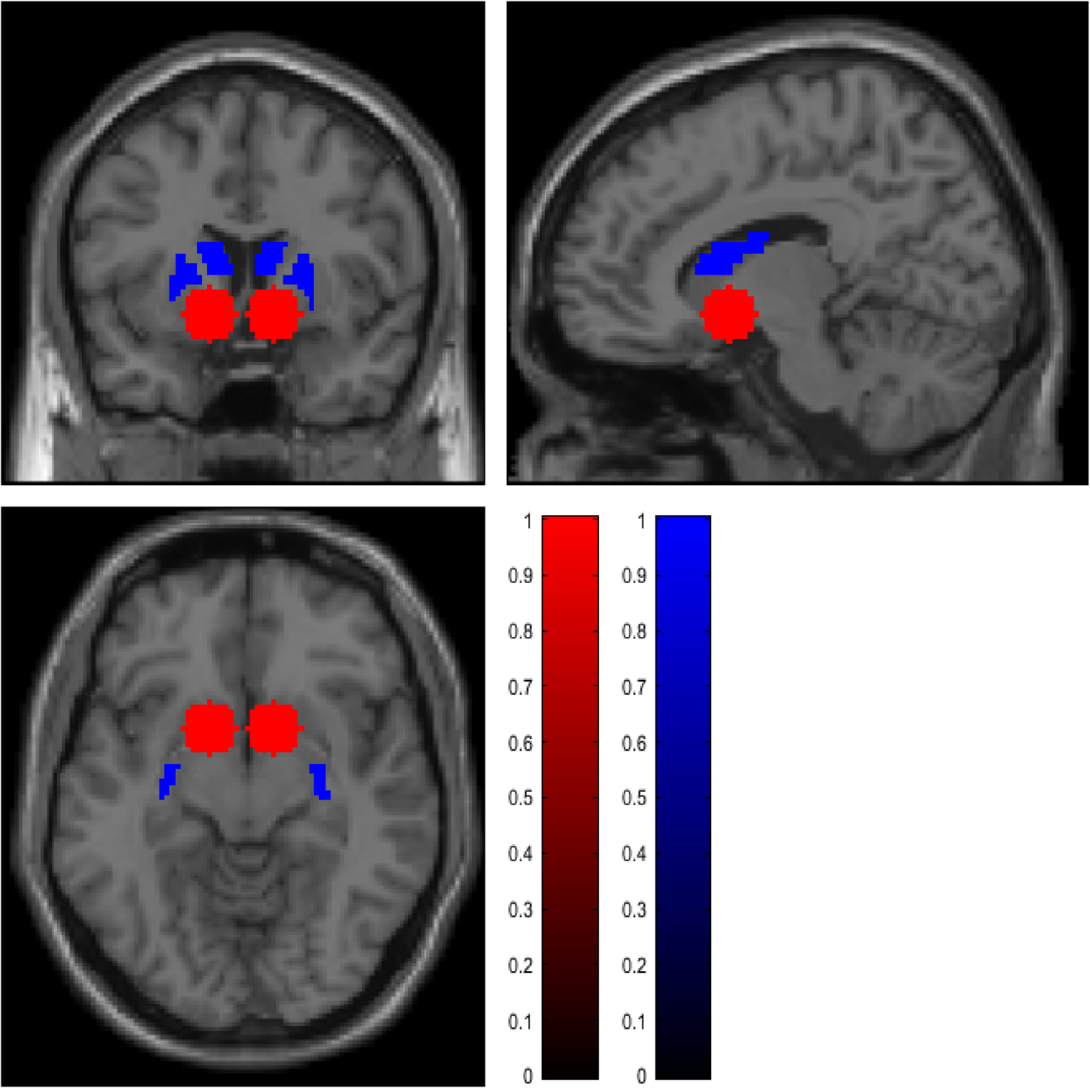
Masks for region of interest (ROI) analysis, ventral striatum (red) two 10 mm spheres bilaterally on the MNI coordinates [+ − 12, 8, − 8]; the dorsal striatum (blue) self-created and already established mask of Vollstädt-Klein et al. (2010)

Individual contrast images described above of the participants were included in a second level analysis (full factorial model) to identify the main effects as well as differences between the verum and the placebo condition. The sequence of drug administration (placebo first/nalmefene first) was considered as a covariate of no interest in the analysis. We conducted a region of interest (ROI) analysis because of the strong a priori hypotheses previously defined in the study protocol (clinicaltrials.gov, NCT02372318) of cue-induced activation of the ventral (VS) and the dorsal striatum (DS) especially due to previous findings of reduce brain activity in mesolimbic regions after intake of opiate antagonists and cue exposure (Myrick et al. 2008; Quelch et al. 2017). The ROI mask for the ventral striatum was created by placing two 10 mm spheres bilaterally on the MNI coordinates [+ − 12, 8, − 8]. These coordinates were determined using a term-based meta-analyses search at the platform www.neurosynth.org. For the dorsal striatum, a self-created and already established mask of Vollstädt-Klein et al. (2010) was used (see Fig. 2). For the ROI analyses, FWE-corrected *p*-values are reported at cluster level.

In addition to the ROI analysis, we conducted a whole brain analysis for exploratory analysis. To control for multiple statistical testing, the probability of a family wise error (FWE) was set to .05. For this purpose, we used the AlphaSim (3dClustSim) method. A voxel wise threshold of *p* < .02 was combined with a cluster extent threshold of 795 (placebo, contrast favorite drink > neutral), 882 (nalmefene, contrast favorite drink > neutral), and 841 voxels (placebo > nalmefene, contrast favorite drink > neutral), determined by the AlphaSim using 25000 Monte Carlo simulations for the whole brain analysis of the ALCUE task. Estimation of smoothness based on the residual images was conducted using SPM by taking the maximum of the 3 estimated parameters in x, y, and z directions.

The self-reported craving was analyzed in SPSS (Statistical Package of the Social Sciences, version 24; SPSS; IBM Corporation, Armonk, NY, USA) using paired *t*-tests.

## Results

Twenty-three participants were randomized to one of the two groups; one group was administered nalmefene at the first time point, and in the other one, nalmefene was given at the second time of measurement. Of the 23 randomized participants, ten participants (44%) reported adverse side effects (five participants of group 1 and five participants of group 2). A mean of 5.8 (*SD* = 2.7) symptoms occurred and lasted a mean time of 37.5 h (*SD* = 22.3 h). For a detailed overview of the adverse side effects that occurred, see Supplement Table 1, Reported side effects.

Five individuals had to be excluded from the analyses because of either withdrawal of informed consent (in most cases due to adverse side effects of nalmefene, e.g., insomnia, vertigo, and nausea, *n* = 4 from group 1) or unsuitability for fMRI scanning (*n* = 1, metal implant). Therefore, the final sample consisted of 18 participants who finished the whole experimental procedure. Because of technical problems (*n* = 1), or too much head movement (*n* = 2), only 15 participants were included in the final fMRI-data analysis. However, the sample of the 18 participants was used to evaluate the behavioral data. Of these 18 participants, 12 were male (67%), and 10 of them were smokers (59%). The mean age was 50.3 years (*SD* = 13.9 years), and a mean of 6.4 (*SD* = 1.4) DSM-5 criteria for alcohol dependence was met. On average, participants consumed 6.4 (*SD* = 4.3) standardized alcoholic beverages (12 g each per drink) per day, for a mean amount of 76.9 (*SD* = 52) grams per day. In the 90 days before the baseline examination, participants had an average of 54 (*SD* = 31.6) heavy drinking days. On the ADS, participants scored an average of 8.7 (*SD* = 4.4). For the extent of harmful use and alcohol dependence (AUDIT), the mean score was 17.2 (*SD* = 5.8). For detailed demographics information of these 18 respectively and 15 participants, see Table 1.

**Table 1:** Effects of single-dose nalmefene on cue-reactivity and craving in alcohol use disorder

	Investigation completed (*n* = 18)			Cue-reactivity task completed (*n* = 15)		
	*M*	*SD*	*n* (%)	*M*	*SD*	*n* (%)
Age	50.3	13.9		52.3	14.1	
Sex						
Male			12 (67%)			10 (67%)
Female			6 (33%)			5 (33%)
Smoker			10 (56%)			7 (47%)
Drinks per day^a^	6.4	4.3		6.8	4.6	
Amount of pure alcohol per day (grams)^b^	76.9	51.8		81.1	55.3	
Abstinent days^c^	21.2	22.9		20.4	22.2	
Heavy drinking days (> 48 g, female/> 60 g, male)^c^	54.0	31.6		53.8	31.1	
Number of fulfilled DSM-5 criteria for AUD	6.4	1.4		6.4	1.4	
ADS	8.7	4.4		8.5	4.7	
AUDIT	17.2	5.8		17.4	6.1	
BDI	6.2	6.1		6.1	6.2	
FTND^d^	3.4	3.0		3.3	2.7	

*Notes. M* mean, *SD* standard deviation, *ADS* Alcohol Dependence Scale, *AUDIT* Alcohol Use Disorders Identification Test, *BDI* Beck Depressions Inventory, *FTND* Fagerström Test for Nicotine Dependence

^a^12 g per drink

^b^Conversion formula: amount in ml * (Vol.-%/100) *0.8 = gram pure alcohol

^c^Refers to the 90 days before baseline

^d^Refers to the subgroup of smokers (*n* = 10 resp. 7)

### fMRI results

**Fig. 3:**
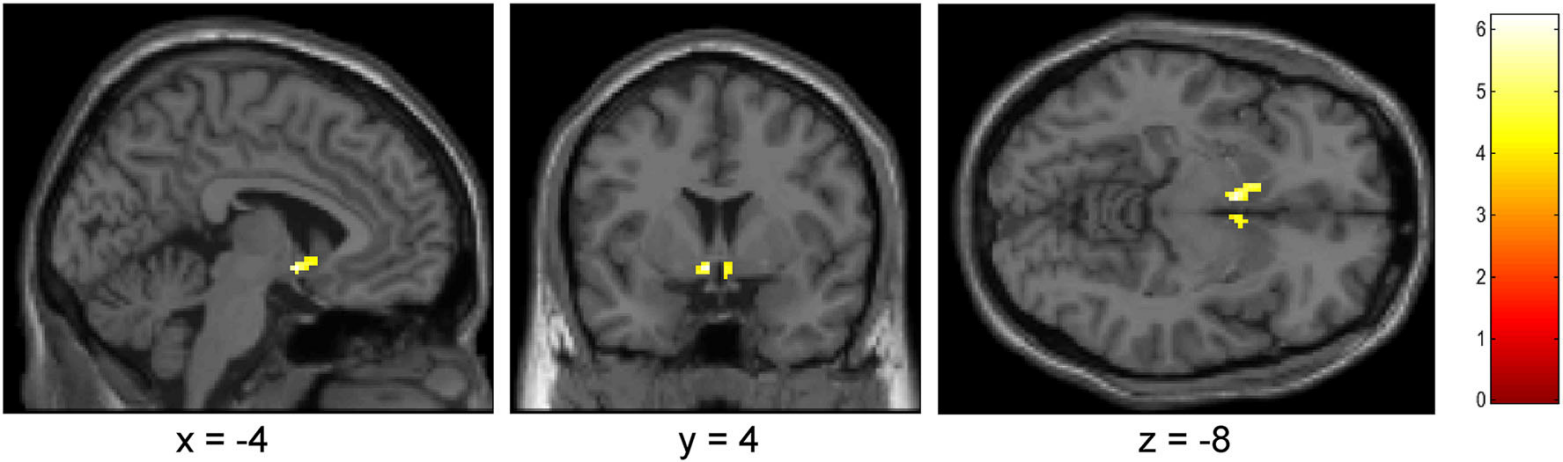
Results of ROI analysis for ventral striatum; *p* < .001 (uncorr), 10 voxel, contrast: placebo > nalmefene, alcohol (favorite) > neutral

Overall, data of 15 heavy drinkers was compared between the conditions nalmefene and placebo. There was a significant lower neural cue-reactivity (favorite drinks > neutral) in the ventral striatum (a priori ROI) in the nalmefene condition compared to the placebo condition [(x,y,z) = (− 4, 4, − 8), *t* = 6.20, *p*_*FWE corrected*_ = .007; (x,y,z) = (4, 4, − 10), *t* = 4.50, *p*_*FWE corrected*_ = .032]; see Fig. 3. Such a difference could not be observed for the dorsal striatum. For the ventral striatum, the actual data demonstrated an effect size of *d* = 0.74 resulting in a power of 86%. Aside from that, there was no main effect for the contrast alcohol (fav) > neutral in the ROI analyses for placebo or nalmefene condition.

However, the explorative whole brain analysis (i.e., non-ROI-based approach) revealed a broadly activated network (for the contrast favorite drink > neutral) including striatal areas, limbic regions (hippocampus and anterior cingulate), and inferior and middle frontal gyrus under placebo, but not under the nalmefene condition (see for a detailed overview Supplement material Fig. 3 and Supplement Tables 2 and 3).

In addition, the exploratory whole brain analysis for the direct comparison placebo vs. nalmefene showed an interaction effect, driven by a decreased activation under nalmefene (i.e., placebo > nalmefene and favorite drink > neutral) in middle frontal gyrus, anterior cingulate, postcentral gyrus, superior frontal gyrus, and middle temporal gyrus (see in detail Supplement Table 4). There was no significant increase in brain activation in any brain region by nalmefene.

### Subjective craving data (behavioral data)

**Fig. 4:**
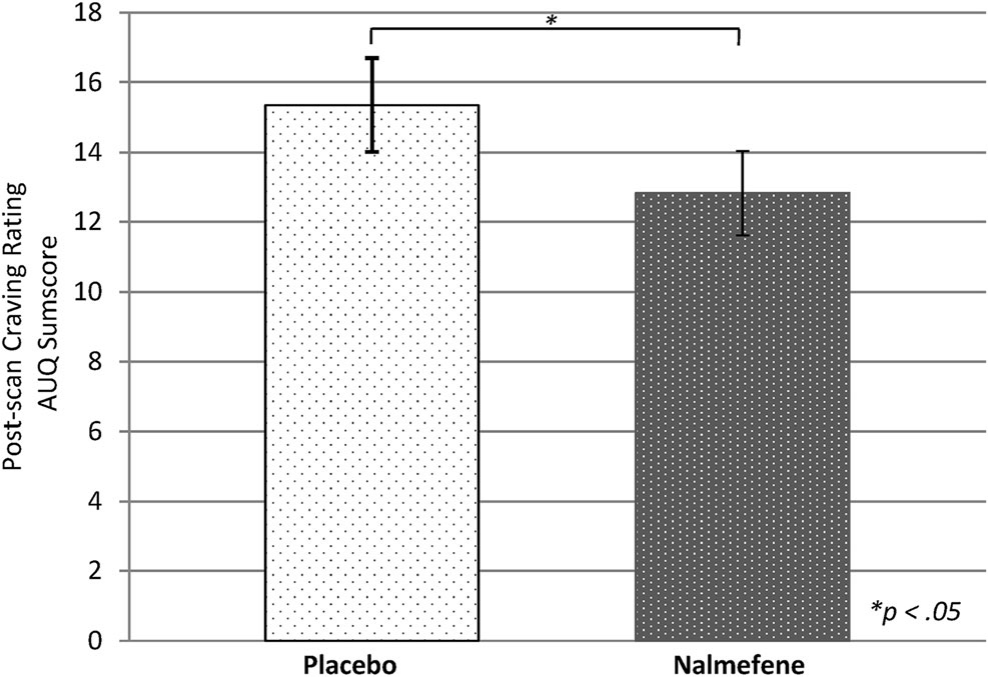
Comparison sum score of AUQ after fMRI cue-reactivity task between nalmefene and placebo

No significant difference in pre-scanning AUQ or ACQ craving between the nalmefene and placebo condition was present. However, participants reported significantly lower cue-induced craving (measured by AUQ) directly after the ALCUE fMRI task in the nalmefene compared to the placebo condition (*t* = 1.79, *p* = 0.046; mean ± SD nalmefene: 12.82 ± 5.02 [range: 8–38], placebo: 15.35 ± 5.52 (range: 8–24); *d* = 0.479, *r* = 0.41; see Fig. 4). For the ACQ craving, no such difference could be observed after the fMRI session (*t* = 0.66, *p* = 0.26; mean ± SD nalmefene: 42.47 ± 17.08 and placebo: 45.12 ± 19.81). An overview of the raw values can be found in Supplement Table 5, Means and standard deviations of the ACQ and AUQ*.*

Regarding the alcohol craving measured during the cue-reactivity task using a VAS ranging from 0 (no craving) to 100 (extremely extensive craving), we found no significant differences between the drug conditions neither for the craving after favorite alcohol cues (*t* = 0.65, *p* = 0.53; mean ± SD placebo: 26.46 ± 29.51 and nalmefene: 22.45 ± 28.45) nor after neutral cues (*t* = − 1.01, *p* = 0.33; mean ± SD placebo: 08.83 ± 14.98 and nalmefene: 10.96 ± 12.98). Also the difference score (favorite alcohol cues − neutral cues) did not differ significantly under either the placebo (mean ± SD: 17.63 ± 21.17) or the nalmefene (mean ± SD: 11.49 ± 20.87) condition, *t* = 1.04, *p* = 0.32.

Linear correlations between questionnaires and VS and DS activations and linear correlations between difference scores (verum versus placebo condition) were examined. There were no significant correlations between (changes in) neural cue-reactivity responses and (changes in) craving.

## Discussion

To the best of our knowledge, we are the first to show that a single-dose nalmefene reduces the neuronal response to alcohol associated stimuli in the ventral striatum. Nalmefene seems to influence neuronal brain responses, responsible for reward-associated behavior (Schultz et al. 1997; Wrase et al. 2007) and may affect substance-related behavior consequently. Such an effect was not revealed for the dorsal striatum. In addition, we could show a reduced subjectively reported craving measured by AUQ after a cue-reactivity task under nalmefene compared to placebo.

This finding expands the results of a first fMRI study by Quelch et al. (2017) who demonstrated similar to the present investigation decreased brain activation in the mesolimbic system under the influence of nalmefene during reward anticipation. In contrast to the present study, Quelch et al. (2017) used a monetary incentive delay task during an intravenous alcohol challenge. We on the other hand administered nalmefene 2 h before the presentation of alcohol-related stimuli respectively alcohol consumption according to a preventive approach as recommended in the European public assessment report (EPAR) for Selincro® (Online document. Online document. European Medicines Agency [EMA] 2013). This naturalistic approach reflects the use of nalmefene as medication for the reduction of alcohol consumption during confrontation with alcohol-associated stimuli.

Furthermore, one possible explanatory approach for the reduced brain activation in the ventral striatum but not in the dorsal striatum after the administration of nalmefene could be an earlier stage of alcohol use disorder in the examined sample. The participants scored an average of 8.7 (*SD* = 4.4) on the ADS (Skinner and Allen 1982). This mean score corresponds to the cut off proposed by Ross et al. (1990) indicating alcohol dependence. For the extent of harmful use and alcohol dependence (AUDIT; Reinert and Allen 2002), the mean score was 17.2 (*SD* = 5.8), indicating a high level of alcohol-related problems (cut-off > 16, Babor et al. 2001). It has been hypothesized that a “ventro-dorsal shift” characterizes the switch from “choice to habit” on a neural basis mirroring the progression of the alcohol dependence (Everitt and Robbins 2005; Everitt and Robbins 2016; Vollstädt-Klein et al. 2010). In the present study, all participants met DSM-5 criteria for AUD. However, none of the participants received any previous (semi-) inpatient detoxification treatment. In addition, in the present sample, the mean ADS score and an average of 81 g (*SD* = 55.3) of consumed pure alcohol per day may indicate that the investigated individuals are “between” light and heavy drinkers and therefore are not yet fully habitual- but rather reward-motivated. This might suggest that especially individuals in early stages of alcohol use disorder could benefit from an add-on therapy with nalmefene. Thus, the results found here suggest that nalmefene could be an agent supporting regaining control over substance use, especially at an early stage of substance use disorder. This approach to regaining control in early stages of substance use disorders (SUD) needs further investigation because it has not yet been sufficiently examined (Heinz et al. 2020).

In a further analysis of Vollstädt-Klein et al. (2019), we investigated the effect of nalmefene on neural activation using fMRI during the presentation of emotional faces pictures in this sample. Results of this suggest that nalmefene is able to influence neuronal processes responsible for social skills and empathy. According to this, nalmefene could have additional useful effects, in addition to its anti-craving effect, especially in the social context, which in turn could support the reduction in amount of drinking (Vollstädt-Klein et al. 2019).

Intriguingly and complementing the above described findings, previous studies have shown that increased activation of the ventral striatum by alcohol-associated cues was associated with an increased risk of relapse in alcohol-dependent individuals. This also suggests that cue-induced activation of ventral striatum might increase the risk of relapse (Braus et al. 2001; Grüsser et al. 2004; Heinz et al. 2009). Against this backdrop, it has to be emphasized that a reduction of the cue-induced brain activation in the ventral striatum through nalmefene might reduce the risk of relapsing into severe drinking patterns.

For the structurally similar opiate antagonist naltrexone, a reduced neuronal activity on alcohol stimuli in the ventral striatum could be demonstrated by fMRI investigation likewise (Myrick et al. 2008) which is also expressed on the behavioral level. Treatment with naltrexone reduces both subjectively craving and the amount of consumed alcohol compared to placebo treatment (Hendershot et al. 2016; Rösner et al. 2010).

Moreover, in the present study, a significantly lower self-reported craving, as measured with the AUQ, after the fMRI scan was revealed in the nalmefene condition, compared to placebo. This finding could be based on the fact that the AUQ is useful to capture substance cravings before and after a cue-reactivity task (MacKillop 2006), and that this questionnaire is a quick self-report instrument (< 1 min) to assess craving with high internal consistency and high test-retest reliability (Drobes and Thomas 1999). In addition, the AUQ was shown to correlate significantly positively with alcohol dependence severity and with scores on the Obsessive Compulsive Drinking Scale indicates construct validity (Bohn et al. 1995). Also, de Laat et al. (2019) reported significantly reduced alcohol craving as measured by AUQ after naltrexone administration which was also associated with kappa opioid receptor (KOR) availability. This is also in line with our finding, due to a partial agonistic activity at the kappa opioid receptor of nalmefene. Taking together, the reported finding might indicate that nalmefene is able to reduce craving and thus the hedonic effect of alcohol. The particular strength of the within-subjects study design is that each participant serves as their own control and thus reduces the error variance (Bakeman and Robinson 2005).

On the other hand, not all craving measurements showed significant reduction by nalmefene. An explanation for low and not significant different craving measured by ACQ and the fMRI task between the two conditions may also be an effect of social desirability. Even if direct questioning about current substance demand is a common approach, this can be distorted by social desirability (Wiers and Heinz 2015). Additionally, many participants reported that the investigation took place at a time (in most cases, during the day) that was outside of their usual time to drink (in the evening). This aspect in combination with the fact that the participants were in an examination situation could have additionally influenced the self-reported craving. In addition, the ACQ measures the aspect of loss of control relatively insufficiently, so that a multidimensional craving assessment is recommended by combining it with other measures such as the OCDS (Raabe et al. 2005). Future studies could use additional (indirect) measurements of physiological outcomes, such as skin conductance or heart rate, to support the measurement of craving (Drobes and Thomas 1999). Moreover, this could be useful as there is evidence that not all individuals can consciously perceive their substance craving (Tiffany and Conklin 2000), which could be problematic when using self-report measures.

Unexpectedly, there was no main effect for the type of stimulus in the ROI analysis, i.e., there was no increased striatal activation during favorite alcoholic stimuli under the nalmefene or the placebo condition. However, the explorative whole brain analysis with a more liberal threshold indicated that alcohol stimuli induced increased activation compared to neutral stimuli in the ventral and dorsal striatum. Here, also other relevant brain regions implicated in cue and reward processing as middle frontal gyri and anterior cingulate cortex (Lukas et al. 2013; Schacht et al. 2013) were shown to be increased in the whole brain analysis under placebo and reduced by nalmefene.

In addition to the lack of observable main effects in the ROI analysis for the type of stimulus, the small sample size should be mentioned as a further weakness of this study. On this account, small effects may have been lost. In addition, the type of stimuli may not be suitable for every participant to provoke cue-reactivity (Mucha et al. 2008). Although we analyzed the contrast “favorite drink > neutral,” the picture stimulus set might not have been optimal for each participant.

Another limitation in this specific sample is an unequal distribution of participants between the two groups (see Fig. 1 and group 1: *n* = 5, group 2: *n* = 10). This is partly explained by dropout due to adverse side effects (e.g., insomnia, vertigo, and nausea; for detailed description, see Supplement Table 1, Reported side effects). In the group of participants who received the nalmefene at the first time point (group 1), four participants dropped out because of adverse side effects, while in group 2 (placebo T1, nalmefene T2), no dropout after the first time point occurred (see Supplementary Figure 1 CONSORT flow diagram). Therefore, drop-out might be affected by medication, which could limit the results as an influence of the sequence of drug administration.

Nevertheless, further research is needed to assess long-term effects of nalmefene on neuronal response since even non-medical interventions like cue-exposure therapy led to reductions in cue-reactivity (Mellentin et al. 2017).

Taken together, nalmefene attenuated alcohol cue-reactivity in the ventral striatum of non-treatment-seeking AUD patients compared to placebo and reduced subjective alcohol craving in our study, supporting the anti-craving effect of this drug. So our findings support the assumption that nalmefene might reduce the amount of drinking via attenuation of neural alcohol cue-reactivity.

As previous studies suggested that especially individuals with a high cue-reactivity in the ventral striatum (Mann et al. 2014; Schacht et al. 2017) might particularly benefit from treatment with opioid-antagonists, future studies could establish predictors for successful treatment with nalmefene.

## Supplementary Information

ESM 1 (DOCX 1750 kb)
