# Supplementary material for: Nalmefene attenuates neural alcohol cue-reactivity in the ventral striatum and subjective alcohol craving in patients with alcohol use disorder
Source: Psychopharmacology (Berl). 2021 Apr 12;238(8):2179–89. doi: 10.1007/s00213-021-05842-7 (PMC8292278; doi:10.1007/s00213-021-05842-7)
Supplement: Supplementary file 1 — (DOCX 1750 kb) [file 213_2021_5842_MOESM1_ESM.docx]

**Supplementary Material**

**Supplementary Figures**


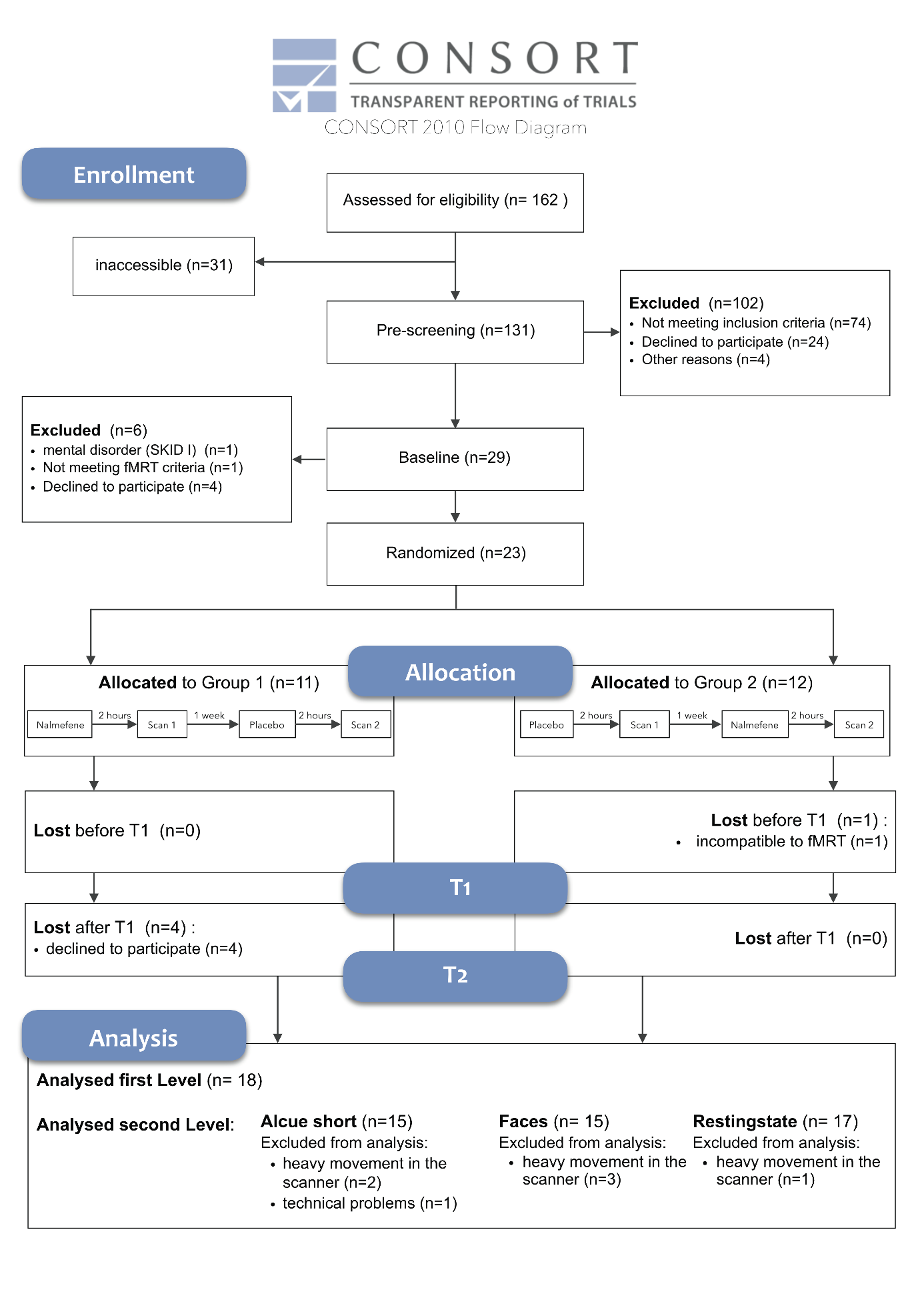


**Supplementary Figure 1:** CONSORT flow diagram displaying the enrollment, allocation and analysis process

**
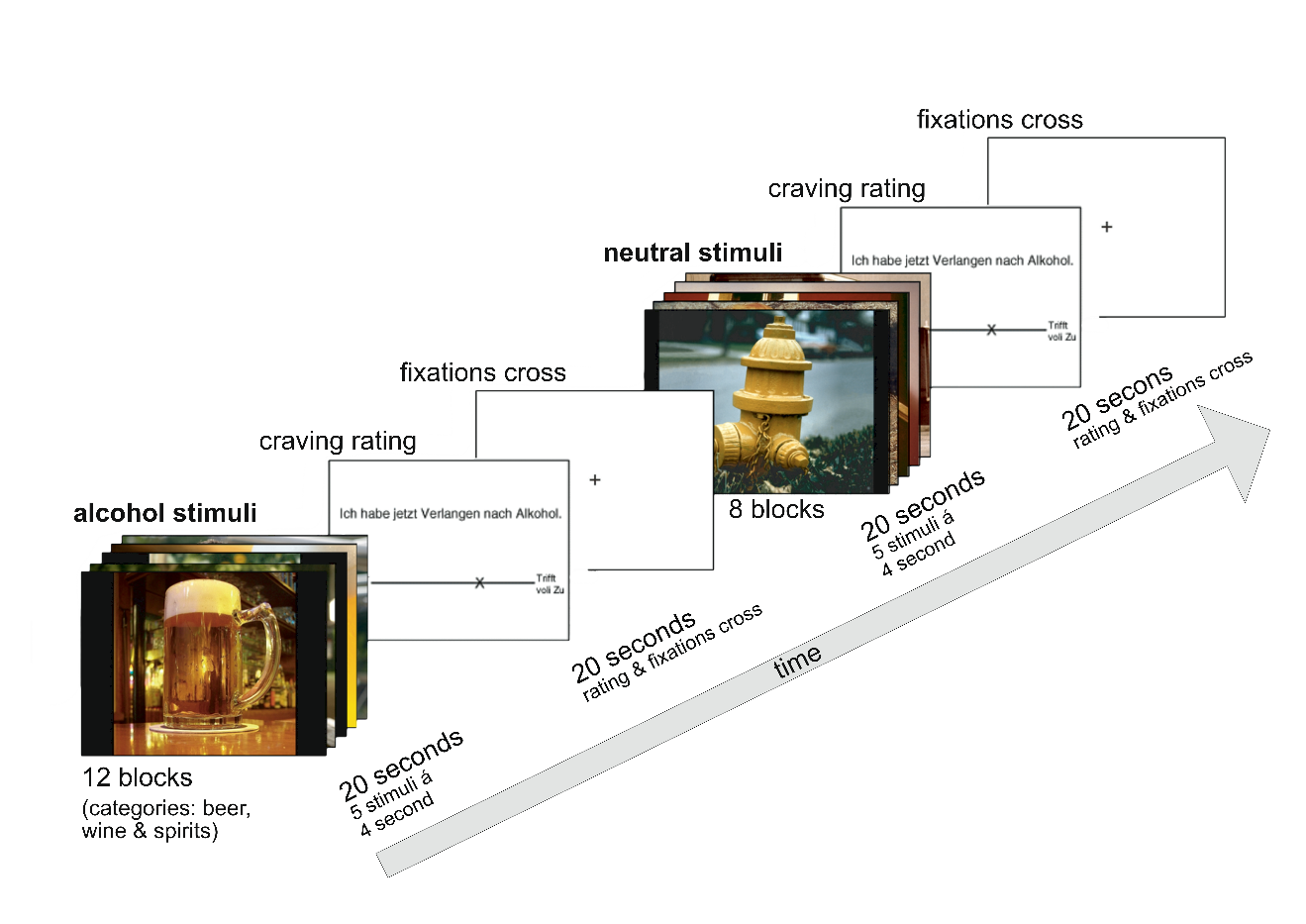
**

**Alloction**

**Supplementary Figure 2:**  Functional magnetic resonance imaging task, block design including alcohol and neutral pictures as well as a visual analog scale (VAS) for craving ratings (Alcue, Vollstädt-Klein et al., 2010)

**
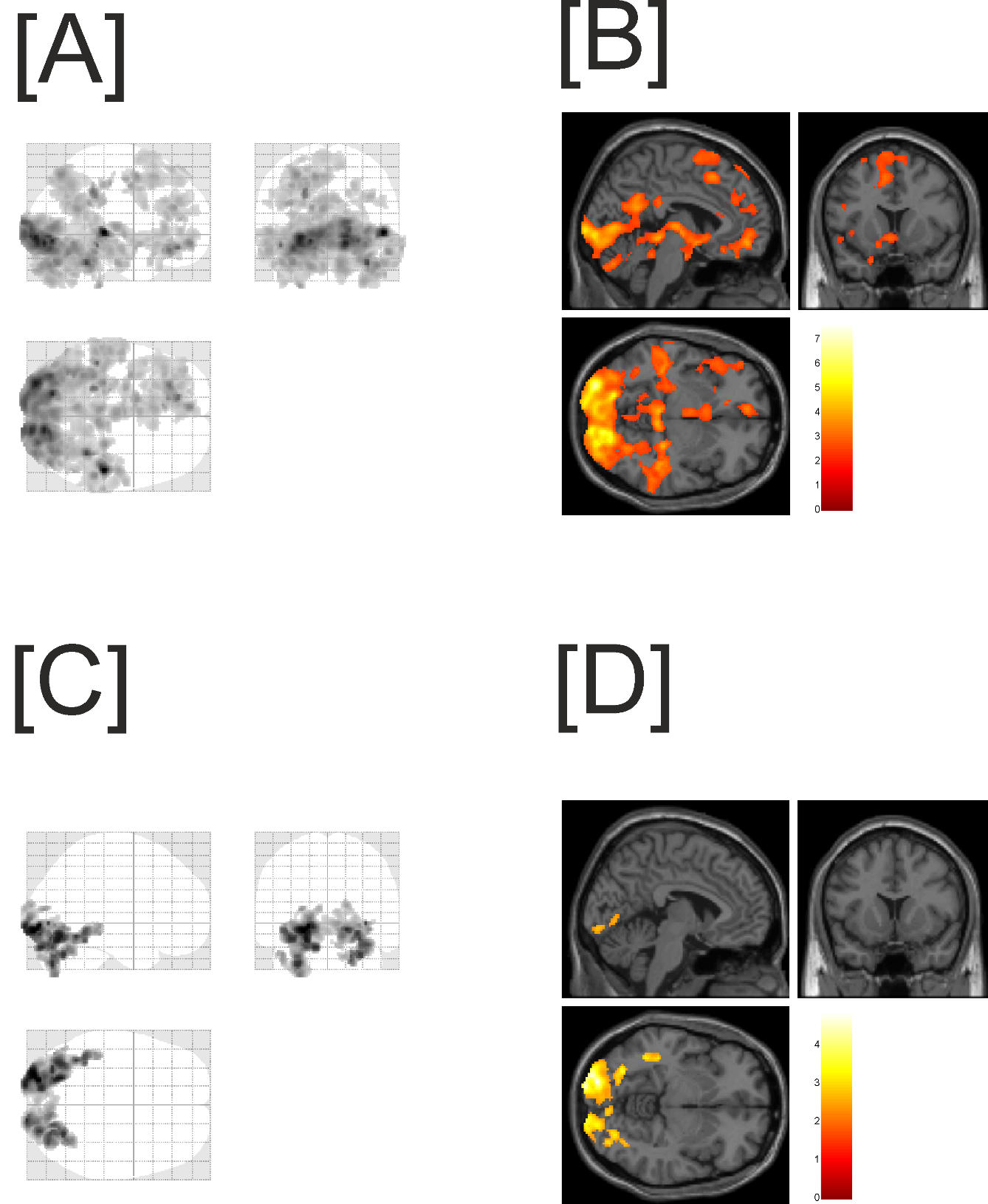
**

**Supplementary Figure 3:**  Results of whole brain analysis, contrast favorite drink >neutral; [A] & [B] after placebo; [C] ] & [D] after 18mg nalmefene; coordinates (x, y, z): -4, 12, -6; p < .05 FWE-corrected (Monte Carlo-based simulations, corresponding to cluster extent threshold p<.02, 841 voxels)

| Supplement Table 1  *Reported side effects of nalmefene of N=23 and N=15 (included in the final analyses)* | | |
| --- | --- | --- |
| *Symptom* | *N = 23 (%)* | *N = 15 (%)* |
| Insomnia | 5 (22 %) | 4 (27 %) |
| Vertigo | 5 (22 %) | 3 (20 %) |
| Nausea | 4 (17 %) | 2 (13 %) |
| Faintness/drowsiness/tiredness | 4 (17 %) | 4 (27 %) |
| Appetite loss | 3 (13 %) | 3 (20 %) |
| Headache | 2 (9 %) | 1 (7 %) |
| Tunnel vision | 2 (9 %) | 2 (13 %) |
| Tenseness | 2 (9 %) | 2 (13 %) |
| Restlessness | 2 (9 %) | 1 (7 %) |
| Irritability/aggressiveness | 2 (9 %) | 1 (7 %) |
| Perceptional disturbance | 2 (9 %) | 1 (7 %) |
| Attention deficit/lack of concentration | 2 (9 %) | 2 (13 %) |
| Depersonalization | 2 (9 %) | 0 (0 %) |
| Derealization | 1 (4 %) | 0 (0 %) |
| Skin tingle | 1 (4 %) | 1 (7 %) |
| Numbness (of body parts) | 1 (4 %) | 1 (7 %) |
| Body perceptional disturbances | 1 (4 %) | 1 (7 %) |
| Arrest of thought | 1 (4 %) | 0 (0 %) |
| Stimulus satiation | 1 (4 %) | 1 (7 %) |
| Prolonged reaction time | 1 (4 %) | 0 (0 %) |
| Panic/anxiety | 1 (4 %) | 1 (7 %) |
| Diarrhea | 1 (4 %) | 1 (7 %) |
| Dry throat | 1 (4 %) | 1 (7 %) |
| Increased salivation | 1 (4 %) | 1 (7 %) |
| Tremor | 1 (4 %) | 1 (7 %) |
| Palpitation | 1 (4 %) | 1 (7 %) |
| Hot and cold feeling | 1 (4 %) | 1 (7 %) |
| Cold sweat | 1 (4 %) | 1 (7 %) |
| Hyperhidrosis | 1 (4 %) | 1 (7 %) |
| Nervousness/uneasiness | 1 (4 %) | 1 (7 %) |
| Impairment in daily life | 1 (4 %) | 1 (7 %) |

| Supplement Table 2  *Brain regions with increased BOLD-Signal during the cue-rectivity task (contrast favorite drink >neutral) after placebo; p<.05 FWE-corrected (corresponding to cluster extent threshold p < .02, 795 voxels)* | | | | | |
| --- | --- | --- | --- | --- | --- |
| **Lobe** | **Side** | **Brain areas** | **Cluster size in voxels** | **MNI coordinates**  **(X, Y, Z)** | **T_max_** |
| ***Cluster 1*** |  |  |  |  |  |
| Occipital | R+L | Lingual gyrus, cuneus, middle and inferior occipital gyrus, calcarine sulcus, precuneus | 24699 | 50, -30, 0 | 7.62 |
| Temporal | R+L | Fusiform gyrus, superior, middle and inferior temporal gyrus |  |  |  |
| Parietal | L | Superior and inferior parietal gyrus |  |  |  |
| Limbic | L | Posterior cingulate, hippocampus |  |  |  |
| ***Cluster 2*** |  |  |  |  |  |
| Frontal | L | Inferior frontal gyrus (pars orbitalis, triangularis), ventromedial prefrontal cortex (medial OFC), middle frontal gyrus | 3231 | -14, 40, -14 | 4.97 |
| Limbic | L | Anterior cingulate |  |  |  |
| Insular | L | Insula |  |  |  |
| ***Cluster 3*** |  |  |  |  |  |
| Frontal | R+L | Supplementary motor area | 2350 | -14, 2, 50 | 3.97 |
|  | L | Superior and middle frontal gyrus, precentral gyrus |  |  |  |
|  |  |  |  |  |  |
|  |  |  |  |  |  |

| Supplement Table 3  *Brain regions with increased BOLD-Signal during the cue-rectivity task (contrast favorite drink >neutral) after 18 mg nalmefene; p<.05 FWE-corrected (corresponding to cluster extent threshold p < .02, 882 voxels)* | | | | | |
| --- | --- | --- | --- | --- | --- |
| **Lobe** | **Side** | **Brain areas** | **Cluster size in voxels** | **MNI coordinates**  **(X, Y, Z)** | **T_max_** |
| ***Cluster 1*** |  |  |  |  |  |
| Occipital | R | Lingual gyrus, inferior occipital gyrus, fusiform gyrus, calcarine sulcus | 5395 | -20, -92, -6 | 4.84 |
|  | L | Fusiform gyrus, lingual gyrus, middle and inferior occipital gyrus, calcarine sulcus |  |  |  |
|  |  |  |  |  |  |
|  |  |  |  |  |  |

| Supplement Table 4  *Brain regions with increased BOLD-Signal during the cue-rectivity task (contrast favorite drink >neutral) after placebo compared to 18 mg nalmefene (contrast „placebo>nalmefene“); p<.05 FWE-corrected (corresponding to cluster extent threshold p < .02, 841 voxels)* | | | | | |
| --- | --- | --- | --- | --- | --- |
| **Lobe** | **Side** | **Brain areas** | **Cluster size in voxels** | **MNI coordinates**  **(X, Y, Z)** | **T_max_** |
| ***Cluster 1*** |  |  |  |  |  |
| Paretial | R+L | Postcentral gyrus, precental gyrus | 15427 | -52 -42 58 | 6.42 |
| Frontal | R+L | Medial frontal gyrus, precental gyrus, middle frontal gyrus, superior frontal gyrus (medial) |  |  |  |
| ***Cluster 2*** |  |  |  |  |  |
| Temporal | L | Middle temporal gyrus, inferior temporal gyrus, superior temporal gyrus, fusiform gyrus | 3349 | -54, -24, -22 | 5.70 |
| Limbic | L | Hippocampus |  |  |  |
| ***Cluster 3*** |  |  |  |  |  |
| Temporal | R | Middle temporal gyrus, inferior temporal gyrus, fusiform gyrus, | 2658 | 54, -42, -18 | 6.62 |
| Limbic | R | Hippocampus |  |  |  |
| ***Cluster 4*** |  |  |  |  |  |
| Limbic | R+L | Caudate, anterior Cingulate | 1493 | -4, 4, -8 | 6.20 |
| Frontal | L | Superior frontal gyrus |  |  |  |
|  |  |  |  |  |  |
|  |  |  |  |  |  |

| Supplement Table 5  *Means and standard deviations of the ACQ and AUQ* | | | | | | |
| --- | --- | --- | --- | --- | --- | --- |
| Questionnaire | Drug condition | before fMRI | |  | after fMRI | |
|  |  | *M* | *SD* |  | *M* | *SD* |
| ACQ | Placebo | 41.88 | 17.42 |  | 45.12 | 19.81 |
|  | Nalmefene | 41.41 | 13.55 |  | 42.47 | 17.08 |
|  |  |  |  |  |  |  |
| AUQ | Placebo | 13.44 | 5.10 |  | 15.35 | 5.52 |
|  | Nalmefene | 12.18 | 4.71 |  | 12.82 | 5.02 |
| *Notes*. *M* = mean, *SD* = Std. Deviation, ACQ: Alcohol Craving Questionnaire; AUQ: Alcohol Urge Questionnaire. | | | | | | |
